# Extrapulmonary Tuberculosis masquerading as chest wall malignancy: Just never ceases to surprise!

**DOI:** 10.1016/j.idcr.2021.e01114

**Published:** 2021-04-06

**Authors:** Mousa Hussein, Ahmad Abdelhadi, Anam Elarabi, Ibrahim Rashid, Abbas Alabbas, Aisha Aladab

**Affiliations:** a Pulmonary Department, Hamad General Hospital, Qatar; b Internal Medicine Department, Hamad General Hospital, Qatar

**Keywords:** Extrapulmonary tuberculosis, Musculoskeletal tuberculosis, Chest wall mass, Cold abscess, Rib fracture

## Abstract

With the emergence of the acquired immunodeficiency syndrome, we witnessed a higher incidence of disseminated and extrapulmonary tuberculosis. The infection sites commonly include lymph nodes, pleura, and osteoarticular areas, although any organ can be involved. Given the atypical presentation of the extrapulmonary disease, it poses a significant diagnostic challenge for the physicians; therefore, a high index of suspicion should be maintained, particularly where tuberculosis is endemic. Here we present a case of isolated chest wall tuberculosis in an immunocompetent patient.

## Introduction

Tuberculosis (TB) is an infectious disease caused by *Mycobacterium Tuberculosis* (MTB), which spreads through the air and commonly affects the lungs. It has an increased incidence during the last few decades despite the effective management because of the emergence of the human immunodeficiency virus (HIV) and the wide use of immunosuppressive medications [1].

Tuberculosis's most commonly presenting as pulmonary infection, it can affect any organ in the body such as the pleura, central nervous system, lymphatic system, genitourinary system, and musculoskeletal system [2]. One of the rare presentations of the musculoskeletal system infection is chest wall involvement, representing around 2 percent of tuberculosis cases [3]. Herein we present an interesting case of chest wall tuberculosis in a young immunocompetent patient.

## Case presentation

A 24-year-old male patient, who was previously healthy, presented to the Hamad General Hospital complaining of progressive left-sided pleuritic chest pain lasting for a few weeks. He noticed two large lumps in front of his chest, precisely at the same site of the pain slowly increasing in size over four months. He experienced a loss of appetite and weight of eight kilograms over the same duration. The patient denied any history of cough, shortness of breath, fever, or night sweats. He was a lifelong non-smoker, with no contact with sick people and no recent travel, with unremarkable family history.

On examination, he was afebrile, maintaining normal oxygen saturation on ambient air. There were two anterior chest wall masses: the first above the left second rib, measuring 7 × 7 cm, and the second over the left costal margin measuring 10 × 10 cm. The covering skin of both masses appeared normal with no draining sinus or tract. The lumps were mobile, cold, non-pulsatile, and not tender. Chest auscultation and examination of other relevant systems were unremarkable.

His laboratory investigations showed hemoglobin of 11.4 g/dL, C-reactive protein of 65 mg/L, positive Quantiferon TB gold plus, and normal kidney and liver function tests (Table 1).

**Table 1:** Relevant lab investigations.

Investigation	Result	Normal range
WBC count	6.9	4−10 × 10^3/μL
Platelet count	348	15−400 × 10^3/μL
Hb	11	13−17 gm/dL
Creatinine	71	62−106 μmol/L
Sodium	136	136−145 mmol/L
Alanine aminotransferase	31	0−41 U/L
C- Reactive protein	65	0−5 mg/L
Lactate dehydrogenase	178	135−225 U/L
Total protein	78	66−87 gm/L
Albumin	31	35−52 gm\L
Ferritin	436	30−533 μg/L
Quantiferon TB gold plus	Positive	
HIV antigen/antibody ELISA	Non-reactive	

**Fig. 1:**
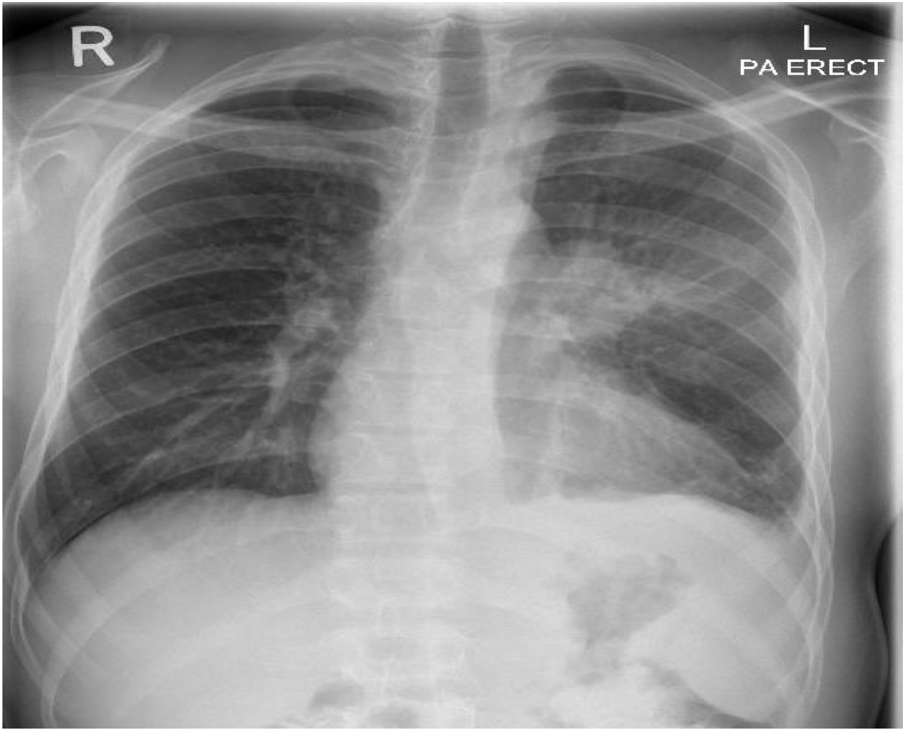
Chest x-ray showed airspace opacity is noted in the left perihilar region.

**Fig. 2:**
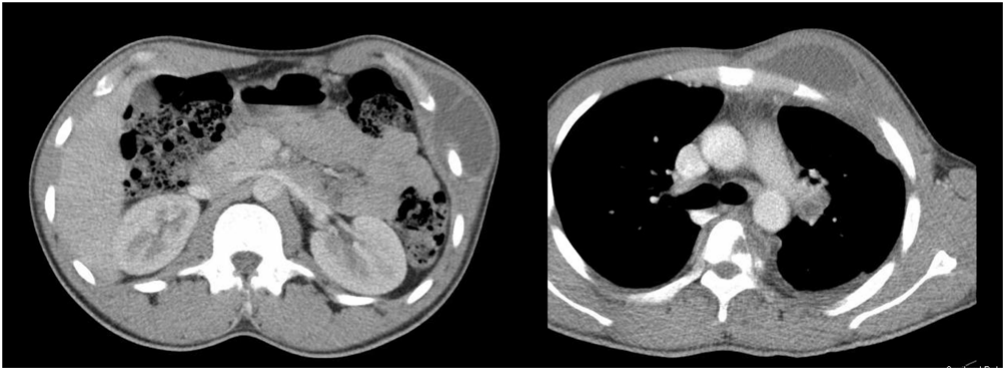
A, B: Two chest wall masses, with enlarged left hilar node showing necrosis.

**Fig. 3:**
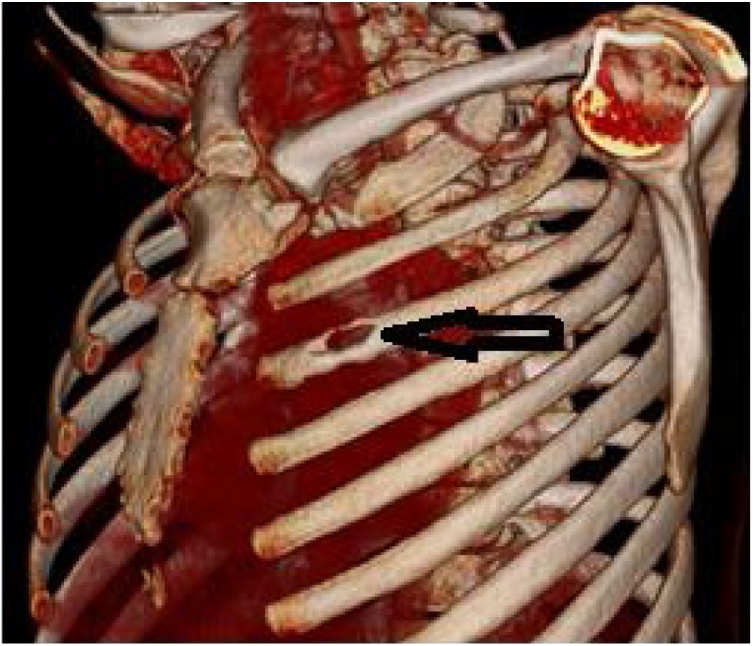
Third rib destruction as shown by the black arrow.

**Fig. 4:**
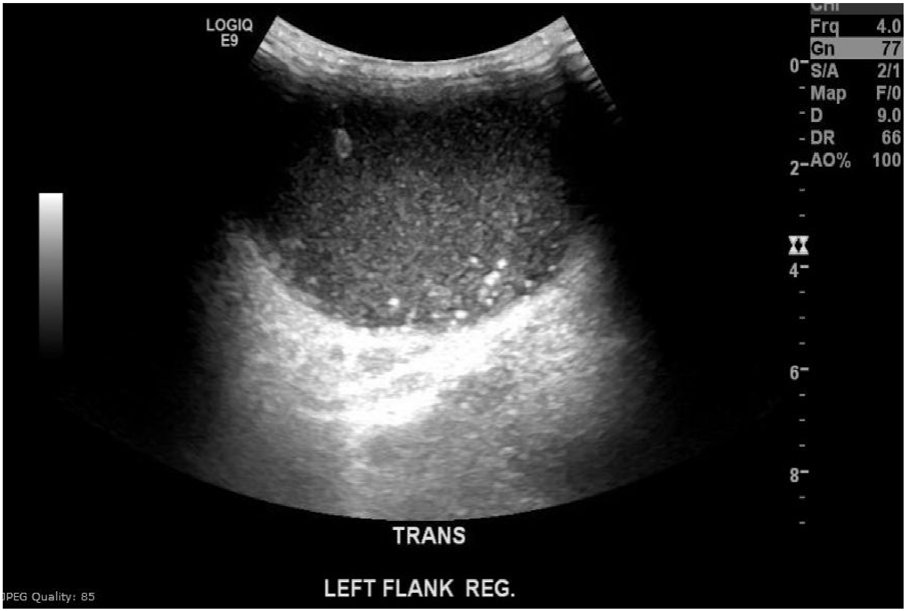
Ultrasound examination of the lesion in the lower chest wall showed well defined hypoechoic area, with varying degrees of internal heterogeneity.

A chest x-ray showed airspace opacity noted in the left perihilar region (Fig. 1). Chest computed tomography (CT) showed two lenticular-shaped collections noted in the left anterior chest wall. One is noted along the internal surface of the left third rib measures 19 × 53 mm causing bone destruction. The other one is in the subcutaneous tissue measures 60 × 33 mm, with an enlarged left hilar lymph node showing peripheral enhancement and central hypodense necrotic area (Fig. 2 A, B) with associated underlying third rib destruction (Fig. 3). Ultrasound examination of the lesion in the lower chest wall showed well defined hypoechoic area, with varying degrees of internal heterogeneity (Fig. 4).

The patient failed to produce any sputum even after induction. The acid-fast bacilli smear (AFB) of the aspirated fluid from the chest wall swelling was negative, but the Polymerase Chain Reaction (PCR) was positive. The aspirated fluid culture grew a pan-sensitive Mycobacterium Tuberculosis Complex (MTB). We diagnosed him with primary chest wall tuberculosis and therefore started on first-line anti-tuberculosis medications. The patient traveled back to Nepal and lost follow up with us.

## Discussion

Tuberculosis is a chronic infectious disease caused by *M. tuberculosis*. Every year, around ten million people are diagnosed with tuberculosis. Despite being a preventable and curable disease, it has been one of the world's leading infectious disease killer [2]. The main risk factors for developing tuberculosis infection are poverty, undernutrition, diabetes, smoking, and immunocompromised conditions, namely, human immunodeficiency virus infection (HIV) [4].

The lungs are the primary site for tuberculosis infection, with various presentations including primary TB, reactivation TB, endobronchial TB, and tuberculoma [4]. The main pulmonary tuberculosis symptoms are fever, cough, difficulty breathing, anorexia, weight loss, and night sweats. However, in the advanced stage and the absence of proper treatment, complications may occur in the form of hemoptysis, pneumothorax, bronchiectasis, and extensive pulmonary destruction [5].

Extrapulmonary tuberculosis refers to TB involving organs other than the lungs (e.g., pleura, lymph nodes, abdomen, genitourinary tract, skin, joints, bones, or meninges). It represented 15 % of the 7.0 million incident cases notified in 2018, and the most typically reported sites were lymph node, pleura, and urogenital tuberculosis [6]. Skeletal tuberculosis accounts for 2–6 % of all tuberculous infections, with the spine being the most commonly affected site. The chest wall involvement and ribs in skeletal tuberculosis are exceptionally uncommon, accounting for < 5 % of bone and joint TB. It is twice more common in males than females, with typical age between 15–30 years, as in our patient [7].

TB abscesses of the chest wall are usually seen at the sternal margins and along the rib shafts. Three mechanisms have been described as responsible for the chest wall involvement, including a direct extension from the pleural or parenchymal disease, hematogenous dissemination, or lymphatic extension [8]. In our case, although the patient did not have any respiratory symptoms with no parenchymal involvement, the hilar and mediastinal lymph nodes enlargement with peripheral enhancement and central necrosis as seen on chest CT scan represents the possible focus for the chest wall involvement.

Chest wall tuberculosis usually presents insidiously as swelling and pain over the bone, with few constitutional symptoms, making the diagnosis difficult and delayed in most cases, averaging 4–28 months. Some patients may present with secondary infection complications, spontaneous fractures of the ribs or sternum, and compression or erosion of the large blood vessels [9]. Differential diagnosis of the chest wall swellings includes granulomatous diseases (sarcoidosis, nontuberculosis mycobacteria), chronic infections (fungal or parasitic), and benign or malignant growth (fibrous dysplasia, osteoblastoma, chondral tumors, malignant bony or cartilaginous tumors, and metastasis) [10]. Our patient presented with four months history of slow-growing and painful anterior chest wall masses, weight loss, and anorexia with no evidence of complications.

The laboratory investigations for diagnosing chest wall TB are non-specific, including slightly raised inflammatory markers (C-reactive protein, white blood cells, and serum ferritin), anemia of chronic disease, which were the same as found in our case [11]. The imaging modalities useful in chest wall TB are radiography, ultrasound, and CT scan. The findings from these modalities help determine the degree of thoracic cage involvement and give a hint for the possible focus of involvement in the form of pleural, parenchymal, and mediastinal lymph nodes abnormalities [12]. Although the Magnetic Resonance Imaging (MRI) of the chest provides a lot of information about the soft tissues and degree of extension of the abscess; CT chest is considered the investigation of choice by many authorsas it is easily accessible, affordable, and provides detailed information almost as MRI chest. Typical CT findings in the chest wall TB include bone destruction, soft tissue masses crossing the fascial planes, with abscess and calcification, and underlying pleuro-parenchymal tubercular involvement [12,13]. There is an increasing role for the ultrasound to help localize the affected areas and the size of the collections. The abscesses usually appeared as hypoechoic areas, with varying degrees of internal heterogeneity. Bone fragments appeared as echogenic foci within these hypoechoic collections [14].

Fine-needle aspiration (FNA) of the chest wall collection represents a simple, non-invasive way to get the diagnosis of tuberculosis. The aspirated material is usually sent for acid-fast bacilli (AFB) smear, PCR, and culture. The yield of AFB smear is low as it needs many bacteria; on the other hand, PCR requires a small number of bacteria and can detect the drug resistance. In addition to identifying the sensitivity to the specific treatment, AFB cultures provide the advantage of knowing the organism species to exclude the *Mycobacteria* other than tuberculosis as a well-known cause of chest wall infections [11,15]. In our patient, PCR and culture confirmed the diagnosis of Mycobacterium Tuberculosis (MTB) with full sensitivity to first-line anti-TB medications.

There is no consensus on the optimal treatment of chest wall tuberculosis. The general role is for the medical treatment, in the form of anti-tuberculosis medications, with a six-month regimen being sufficient in most cases. Surgical intervention may be needed in cases of extensive tissue damage, presence of draining sinuses, or failure of medical treatment [16,17].

## Conclusion

Chest wall tuberculosis is a rare entity that may occur even in an immunocompetent patient. A high index of suspicion is required to make an early diagnosis and overcome the diagnostic dilemma. Using a simple non-invasive procedure in the form of needle aspiration will get the diagnosis in most cases. Early and prompt treatment will prevent the occurrence of complications.

## Author statement

1 Mousa Hussein: Corresponding author, literature review, writing and editing, data collection and analysis.
2 Ahamd Abdelhadi: literature review, following the clinical progression of the case.
3 Anam elarbi: data collection, review the final draft.
4 Ibrahim Rashid: review the final draft.
5 Abbas Alabbas: Management and clinical follow up of the case.
6 Aisha Aladab: review the final draft.

## Declaration of Competing Interest

The authors report no declarations of interest.
